# Comment on the safety of the ultrasound-guided hydrodissection technique for carpal tunnel syndrome

**DOI:** 10.1007/s40477-022-00664-5

**Published:** 2022-02-16

**Authors:** King Hei Stanley Lam, Wai Wah Lai, Ho Yin Ngai, Wing Keung Ricky Wu, Yung-Tsan Wu

**Affiliations:** 1 The Hong Kong Institute of Musculoskeletal Medicine, Unit 305 Telford House, 16 Wang Hoi Road, Kowloon Bay, Kowloon, Hong Kong; 2 Department of Family Medicine, The Chinese University of Hong Kong, Sha Tin, Hong Kong; 3 Department of Family Medicine, The University of Hong Kong, Hong Kong, Hong Kong; 4 Taiwan Association of Prolotherapy and Regenerative Medicine, Taichung, Taiwan; 5 Center for Regional Anesthesia and Pain Medicine, Wan Fang Hospital, Taipei Medical University, Taipei, Taiwan; 6 Department of Physical Medicine and Rehabilitation, Tri-Service General Hospital, School of Medicine, National Defense Medical Center, Taipei, Taiwan; 7 Integrated Pain Management Center, Tri-Service General Hospital, School of Medicine, National Defense Medical Center, Taipei, Taiwan; 8 Department of Research and Development, School of Medicine, National Defense Medical Center, Taipei, Taiwan

Dear editor,

We have read, with great interest, the manuscript by Mathieu et al., entitled “A safe and easy-to-use ultrasound-guided hydrodissection technique for carpal tunnel syndrome: a minimally invasive approach,” J Ultrasound https://doi.org/10.1007/s40477-021-00597-5. Mathieu et al. described the technique protocol to familiarize other doctors with this technique. However, some details in the manuscript brought about concerns.

First, the authors highlighted the use of a sterile transducer cover or sheath. However, when performing ultrasound-guided injection to the nerves, a gel contact medium is not applied between the transducer cover and the skin to optimize its performance and safety. Studies have shown that needles, including those with stylets, carry gel and tissue within the lumen. Thus, the ultrasound gel is delivered around or inside the nerves during regional anesthesia [1]. Pintaric et al. [2] showed that perineural gel injections cause significant inflammation. This inflammation was not due to direct needle trauma, but rather, it was injectate/gel-related. When using a sterile gel as the contact media for perineural injections, the gel carried by the needle further irritates the nerves and causes neurogenic inflammation [2]. For ultrasound-guided procedures, sterile gel is not used as the contact medium between the sheath and the skin. Chlorhexidine gluconate (4%) or povidone-iodine, containing 9–12% available iodine [3], has been used to prevent procedure-related infection. The chlorhexidine-based solutions were more effective than the povidone-iodine (including alcohol-based) [4]. Normal saline solution, a good conduction agent that allows visualization of anatomic structures in ultrasound-guided interventional procedures [5], should be used instead of sterile gel, to prevent potential risks. Therefore, to improve the second step, described in the manuscript, we suggest using chlorhexidine (2%) as the contact medium after sterilizing the mid-forearm to the entire palm and applying a sterile transducer cover.

Steps 4 and 5 of the manuscript mentioned advancing the needle to the inferior and superior surfaces of the median nerve (MN). However, this was not consistent with the basic principle of hydrodissection. Nerve hydrodissection involves using the injectate (“hydro”) to “dissect” or separate the soft-tissues in front of the needle tip. This creates a halo, providing a safe and less painful zone for the needle to follow without coming into contact with, such as blood vessels and nerves, until it reaches the perineurium [6, 7]. This is essential when performing nerve hydrodissection without local anesthetics. This method reduces the pain felt by the patient and prevents damage to other blood vessels and nerves (Fig. 1B).

**Fig. 1:**
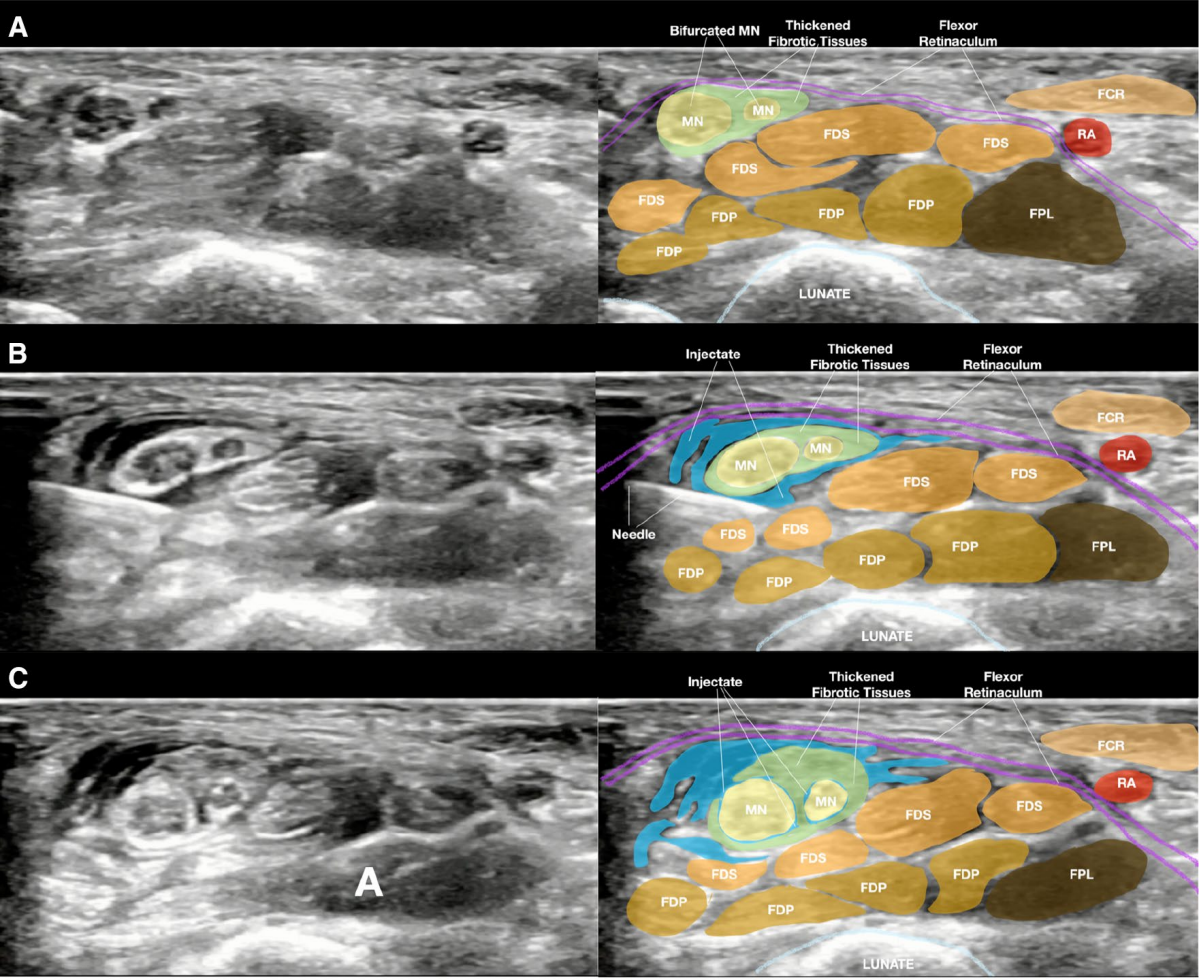
**A** Shows the sonoanatomy of a bifurcated median nerve (MN) (in yellow) in the carpal tunnel entrapped by the thickened fibrotic or scarring tissues (in green). **B** Shows the partially hydrodissected MNs surrounded by injectate (in blue) but the thickened fibrotic or scarring tissues are still trapping the MNs, so that the nerve still looks elliptical. **C** Shows a completely hydrodissected MN with both bundles looked rounded or oval and surrounded by injectate. FCR, flexor carpi radialis tendon; FDS, flexor digitorum superficialis tendon; FDP, flexor digitorum profundus tendon; FPL, flexor pollicis longus tendon; MN, median nerve; RA, superficial palmar branch of radial artery

Third, the authors failed to emphasize that, to achieve optimal results and prevent recurrence, all the fibrotic or scar tissues on the palmar, dorsal, radial, and ulnar aspects of the MN should be separated from the MN. In steps 4–6, the authors injected 1.5 cc of the injectate into the inferior surface of the MN. More than 3 cc was injected to separate the flexor retinaculum from the superior surface of the MN. When a patient develops CTS, the tethering of the fibrotic or scar tissues on all sides of the MN should be freed. The final appearance of the MN should be round or oval, and completely surrounded by a halo (Fig. 1C, Video 1 in ESM). [6, 8] Particularly, patients with tethering from the flexor tendons experience MN entrapment and compression between the flexor tendons and the flexor pollicis longus tendon [9]. As shown in Fig. 6 of Mathieu et al.’s manuscript, the MN maintained an elliptical shape after hydrodissection. Moreover, numerous fibrotic or scarring tissues were noted on the radial, ulnar, and inferior aspects of the MN. This indicated that the MN was still firmly tethered to the flexor tendon sheath. Based on clinical experience, this indicated a partially hydrodissected MN (Fig. 1B).

Lastly, Fig. 7 of Mathieu et al.’s manuscript resembled Fig. 2 of reference 3 of their manuscript. Specifically, they were similar in terms of depicting the left-sided nerve swelling [6]. Additional descriptions were included, and the normal-sized right nerve with the solitary swollen fascicle was removed. The original picture showed that a single needle entry point for two nerves required simultaneous treatment. Since Mathieu et al., removed the right side of the nerve only, leaving the needle pathway was inappropriate and unnecessarily long for an MN hydrodissection in the carpal tunnel. May the authors confirm whether they obtained permission to use a part of the original picture?

This manuscript highlighted that sterile gel was not the best contact medium for ultrasound-guided interventions, especially in cases with nerve involvement. Chlorhexidine in alcohol or normal saline solution was a more effective and safer option. This article also emphasized the crucial techniques of ultrasound-guided nerves hydrodissections. The basic principle of hydrodissection is to inject fluid to push away soft tissues and create a halo in front of the needle. This method creates a safe zone for the needle tip to advance, without coming into contact with significant structures. For instance, the MN in the carpal tunnel is avoided through hydrodissection of the fibrotic scar tissues on the palmar, dorsal, ulnar, and radial aspects of the MN. The final appearance of the nerve should be round or oval and surrounded by a halo.

## Supplementary Information

Below is the link to the electronic supplementary material.

Supplementary file1 (DOCX 67 kb)
