# Supplementary material for: Comment on the safety of the ultrasound-guided hydrodissection technique for carpal tunnel syndrome
Source: J Ultrasound. 2022 Feb 16;26(1):285–7. doi: 10.1007/s40477-022-00664-5 (PMC10063760; doi:10.1007/s40477-022-00664-5)
Supplement: Supplementary file 1 — Supplementary file1 (DOCX 67 kb) [file 40477_2022_664_MOESM1_ESM.docx]

**Comment on the safety of the ultrasound-guided hydrodissection technique for carpal tunnel syndrome**

Video 1.

<https://www.dropbox.com/s/3k9asx7xuwtzady/MN%20HD%20B%20mode%20only%20animation.mp4?dl=0>


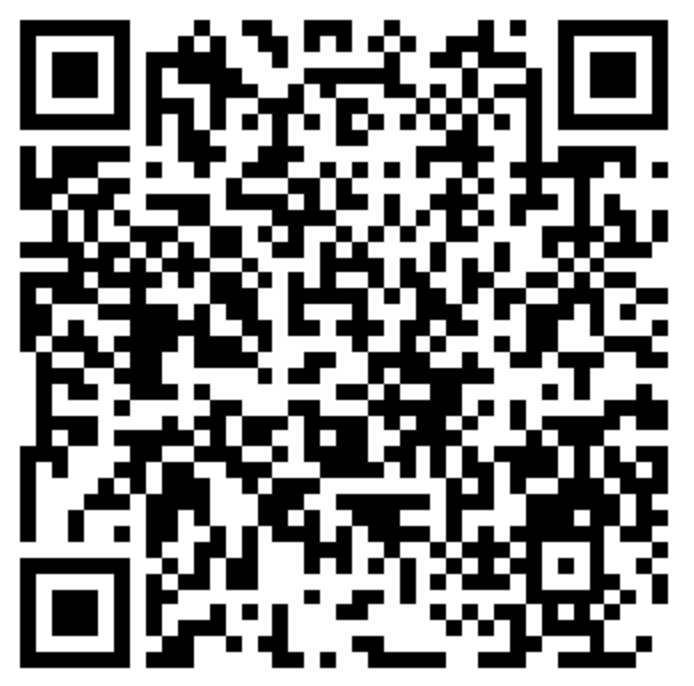


Video shows an ultrasound-guided hydrodissection of the median nerve from the ulnar side, starting with the hydrodissection below the nerve first and then above and with the final appearance of the nerve appearing much more rounded and oval than before hydrodissection and the halo surrounding the whole nerve over the ulnar and radial sides. FCR, flexor carpi radialis tendon; FDS, flexor digitorum superficialis tendon; FDP, flexor digitorum profundus tendon; FPL, flexor pollicis longus tendon; MN, median nerve; RA, superficial palmar branch of radial artery.
